# Supplementary material for: Black Rock City versus Manhattan: An economist’s view
Source: PLoS One. 2021 Jan 11;16(1):e0244331. doi: 10.1371/journal.pone.0244331 (PMC7799750; doi:10.1371/journal.pone.0244331)
Supplement: S1 Appendix — (DOCX) [file pone.0244331.s001.docx]

**Appendix: Derivations**

1. **Population.** Equation (1) in the text describes city population, *N*, as a function of, among other things, the outer edge of the city = the length of an artery = $\bar{u}$. It is the integral of an expression for population on a given iso-cost line, *n*{*u*}, from 0 to $\bar{u}$ (see, e.g., Yinger 2018). The parameter values for the calculations in this paper are *a* = 0.2, *α* = 0.2, *b* = 1/(*aα*), *A* = 0.003, *C* = 0.060629, *t* = 1.5, and $\bar{u}$ = 30. Some calculations in this paper find a city’s *N* holding $\bar{u}$ constant. Some calculations find the change in $\bar{u}$ required to bring one city’s population up to another’s. I approximate this change in $\bar{u}$ using the derivative of Equation (1). To be specific, I rearrange this derivative to find d$\bar{u}$for a given population difference across cities, d*N*:

where *ϕ* stands for the city-specific land constant and the other terms are the same across cities.

1. **Circle City.** The first part of Circle City’s area is the integral of the distances along the circular streets, *ρ*{*u*ʹ}, from the point where the outermost street that circles the CBD, which goes through point C in Figure 2, intersects with the commuting shed to the point where *u*ʹ equals $\bar{u}$.

$$A_{1,Circle}=\int_{n\bar{t}\bar{u}/(\pi+n\bar{t})}^{\bar{u}} \bar{t}(\bar{u}-u')du'=\frac{\pi^{2}\bar{t} \bar{u}^{2}}{2(n\bar{t}+\pi)^{2}}$$

where $\bar{t}=t_{a}/t_{s}$ and *n* is the number of arteries. The second part is the area of the circle defined by the circular street through point *C*, which is:

$$A_{2,Circle}=\left(\frac{\pi}{2n}\right)\left(\frac{n\bar{t} \bar{u}}{\pi+n\bar{t}}\right)^{2}$$

These two formulas refer to one of the 2*n* segments in the city. The area of the circular city overall is therefore *A_Circle_* = (2*n*)(*A_1,Circle_ + A_2,Circle_*).

An expression for the length of an iso-cost line in Circle City begins with an expression for the angle, *θ*, based on Equation (2):

$$\theta\{u'\}=\left(\frac{t_{a}}{t_{s}}\right)\left(\frac{u}{u'}-1\right)=\bar{t}\left(\frac{u}{u'}-1\right)$$

where$\bar{t}=t_{a}/t_{s}$. It follows that in Figure 2 $x\{u'\}=u'\sin\{\theta\} \text{and} y\{u'\}=u'\cos\{\theta\}$ . Hence,

$$\frac{dx}{du'}=\sin\{\theta\}+u'\cos\{\theta\}\frac{d\theta}{du'} \text{and} \frac{dy}{du'}=\cos\{\theta\}-u'\sin\{\theta\}\frac{d\theta}{du'}$$

Using the standard distance formula and the identity that $\sin^{2}\{\theta\}+\cos^{2}\{\theta\}=1$, we find that the distance from A to B in Figure 2, which we symbolize with *δ*, is

$$\delta=\sqrt{\left(\frac{dx}{du'}\right)^{2}+\left(\frac{dy}{du'}\right)^{2}}=\sqrt{\left(\left(\frac{t_{a}}{t_{s}}\right)\left(\frac{u}{u'}\right)\right)^{2}+1}=\sqrt{\left(\bar{t}\left(\frac{u}{u'}\right)\right)^{2}+1}$$

Then

$$\int\delta du'=\sqrt{(\bar{t} u)^{2}+u'^{2}}-\bar{t} u\log\left\{\bar{t} u\left(\sqrt{(\bar{t} u)^{2}+u'^{2}}+\bar{t} u\right)\right\}+\bar{t} u\log\{u'\}$$

(According to Wolfram Integrator, this integral is valid only if $\bar{t}$and *u* are positive, which they must be here. See <https://www.wolframalpha.com/calculators/integral-calculator/> .)

The associated definite integral for the iso-cost line that runs through the vertical artery *u* miles from the CBD is the integral in Equation (A.7) evaluated at *u* and $n\bar{t}\bar{u}/(\pi+n\bar{t})$.

As explained in the text, population is based on the length of the ico-cost line through $\bar{u}$adjusted by a “squish factor” to account for the loss of area associated with low values of $\bar{t}$. This squish factor equals actual area divided by unconstrained area. This unconstrained area has two parts. The first is the value of *A_Circle_* evaluated at $\bar{t}$divided by the comparable value with $\bar{t}$ = 1. The second accounts for the area that would exist if the iso-cost line based on $\bar{t}$ = 1 were extended to the full length of the iso-cost line at $\bar{t}$. Let *d* stand for the difference between these two lengths. Now pivot the dotted lines in Figure 2 downward so that the left-most line coincides with the commuting shed boundary and assume that *d* is the hypotenuse of a triangle with sides defined by Equation (A.5). With this set-up and some basic trigonometry, we can identify this second area, which is a pie slice between the two (pivoted) dotted lines. See Table A.1.

1. **Grid City.** The area and iso-cost length for Grid City can be found with the concepts in Figure A1. (The line CD is only relevant for Diag City.) The angle *θ* comes from the number of arteries—and hence of city segments. With 32 arteries, for example, *θ* = *π*/16. Now let 0B = *y* and BD = *x*. Then $t_{a}(\bar{u}-y)=t_{s}x \text{or} y=\bar{u}-x/\bar{t}$. Moreover, *x/y* = sin{*θ*}/cos{*θ*}. Combining these results yields$y=\bar{u}\bar{t}\cos\{\theta\}/(\bar{t}\cos\{\theta\}+\sin\{\theta\})$ and $x=\bar{u}\bar{t}\sin\{\theta\}/(\bar{t}\cos\{\theta\}+\sin\{\theta\})$. It follows that the area of this segment of the city is (*x*)($\bar{u}$)/2 and the length of the associated iso-cost line segment is $\sqrt{(\bar{u}-y)^{2}+x^{2}}$. Further substitution leads to the length of an iso-cost line:

$$I_{Grid}\{u\}=u\left(\left(\frac{\tan\{\theta\}}{\tan\{\theta\}+\bar{t}}\right)\sqrt{1+\bar{t}^{2}}\right)$$

When (A.9) is used to measure *N*, it is evaluated at $\bar{u}$and multiplied by a “squish factor,” *s*, which is the above area divided by the area of a triangle with base $\bar{u}$and height$(I_{Grid}\{\bar{u}\})/\sqrt{2}$.

1. **Diag City.** Figure A.1 can also be applied to Diag City with one of the arteries as the vertical axis. (This treatment differs slightly from the one in Yinger (1993a) because it places the outer edge of the city, $\bar{u}$, along the diagonal artery, not along the vertical axis. The iso-cost lines in Yinger must be multiplied by $\bar{t}$ to make them comparable to the ones derived here.) In this case, AC indicates the length of the iso-cost line for this segment, 0C = *u*ʹ, 0D = *u*, and BD = BC = *x* (since angle *γ* = 45 degrees by the definition of the street network. Because DC is a street and C is a point on the iso-cost line, DC = *ρ* =$\bar{t}(\bar{u}-u)$. As with Grid City, the angle *θ* comes from the number of arteries. Now consider the triangle 0BC. It has sides *x* = *u*ʹsin{*θ*} and *u* + *x* = *u*ʹcos{*θ*}. Finally, consider the triangle BCD, which has two sides equal to *x*, where *x* = *ρ* cos{45^o^} = *ρ* cos{π/8}. These steps give us four equations in four unknowns: *x*, *ρ*, *u,* and *u*ʹ. With the solutions for these unknowns, we can calculate the area of this segment of the city, (*x*)($\bar{u}$)/2, and the length of the iso-cost line: $\sqrt{(\bar{u}-u-x)^{2}+x^{2}}$. Further substitution leads to:

$$I_{Diag}\{u\}=u\left(\frac{\tan\{\theta\}\sqrt{\left(\sqrt{2}-\bar{t}\right)^{2}+\bar{t}^{2}}}{\sqrt{2}\tan\{\theta\}+\bar{t}\left(1-\tan\{\theta\}\right)}\right)$$

The value of *s* is the above area for this city divided by the area of a triangle with base = $\bar{u}$and height = (*x*)(*I_Diag_*{$\bar{u}$} assuming $\bar{t}$=1)/( *I_Diag_*{$\bar{u}$} with actual value of $\bar{t}$). See Table A.1.

In Figure A.1, point C is on the iso-cost line for both Grid City and Diag City when $(\bar{u}-u)\bar{t}=\sqrt{2}x \text{and} (\bar{u}-u-x)\bar{t}=x.$ Solving both equations for *x* and equating the results indicates that C appears on both iso-cost lines (so that areas are the same) when $\bar{t}=\sqrt{2}-1.$

Also, $\rho=\bar{t}(\bar{u}-u) \text{and} x=\bar{t}(\bar{u}-u).$ Now let AB = $\tilde{u} \text{so} (\bar{u}-u)=(\tilde{u}+x).$ Thus

$$x=BC=\frac{\bar{t} \tilde{u}\cos\{\pi/4)}{1-\bar{t}\cos\{\pi/4)}$$

In triangle ABC, $\tilde{u}=x\tan\{\beta\}=x\sin\{\beta\}/\cos\{\beta\}.$ Now the line between a star shape and a polygon is defined by a right angle for α , which implies that *β = θ*. Plugging this expression for $\tilde{u}$ into (A.11) and evaluating the resulting expression with *β = θ*, leads to:

$$\bar{t}=\frac{\cos\{\theta\}}{\cos\{\pi/4\}\left(\sin\{\theta\}+\cos\{\theta\}\right)}$$

This expression equals $1/\sqrt{2}$ = 0.7071 when *θ* = *π*/4, which corresponds to 4 arteries and 8 segments, and declines with *θ* thereafter. Its value at *θ* = *π*/32, for example, is 0.1268. The polygon shape arises when a city has a value of $\bar{t}$greater than the value in Equation (A.12).

Miles from CBD = *u*

Miles from CBD = *u*

CBD = 0

Commuting Shed Boundary

Figure A.1. Areas and Iso-cost Lines with Grids

A

B

C

D

*θ*

*γ*

*β*

*α*

| Table A1. Key Notation | |
| --- | --- |
| Symbol | Definition |
| *u* | Distance from the city center |
| $\bar{u}$ | Distance from the city center to the city’s outer edge = artery length |
| *I* | Length of transportation iso-cost line |
| *L* | Available land |
| *N* | City population |
| *N** | City population per unit of land |
| $\bar{R}$ | Agricultural rental rate for land |
| *t* | Round-trip commuting cost per mile |
| *b* | Product of *α* (housing exponent in utility function) and *a* (land exponent in housing production function) |
| *D* | Population density |
| $t_{a}$ | Round trip commuting cost per mile along an artery |
| $t_{s}$ | Round trip commuting cost per mile along a street |
| *ρ* | Distance from home to artery along a circular street |
| $u'$ | Distance from a circular street to the city center |
| *θ* | Distance from house to artery along a circular street in radians |
| $\bar{t}$ | $t_{a}/t_{s}$ |
| *x** | x-coordinate where iso-cost line meets a shed boundary |
| *y** | y-coordinate where iso-cost line meets a shed boundary |
| *ψ* | Land constant = *I*{*u*}/*u* |
| *s* | “Squish” factor = loss of population due to loss of area compared to a city with $\bar{t}$ = 1 |
|  |  |
|  |  |

| Table A2. "Squish Factors" for Three City Types | | | | | | | | | |
| --- | --- | --- | --- | --- | --- | --- | --- | --- | --- |
| 4 Arteries | | | | |  | 16 Arteries | | | |
| PARAMETERS | |  |  |  |  |  |  |  |  |
| *t_a_* | 1.500 | 1.500 | 1.500 | 1.500 |  | 1.500 | 1.500 | 1.500 | 1.500 |
| *t_s_* | 6.000 | 3.621 | 3.000 | 1.579 |  | 6.000 | 3.621 | 3.000 | 1.579 |
| $\bar{t}$ | 0.250 | 0.414 | 0.500 | 0.950 |  | 0.250 | 0.414 | 0.500 | 0.950 |
|  |  |  |  |  |  |  |  |  |  |
| SQUISH FACTOR (*s*) | | |  |  |  |  |  |  |  |
| Circle City | 0.37 | 0.57 | 0.65 | 0.97 |  | 0.32 | 0.51 | 0.63 | 0.97 |
| Grid City | 0.34 | 0.54 | 0.63 | 0.97 |  | 0.34 | 0.54 | 0.63 | 0.97 |
| Diag City | 0.23 | 0.41 | 0.52 | 0.97 |  | 0.23 | 0.41 | 0.52 | 0.97 |

Note: These “squish factors” account for the loss of population associated with a loss of area compared to a city with $\bar{t}$ = 1.

Appendix Reference

1. Yinger, John (Editor). *Housing and Commuting: The Theory of Urban Residential Structure*. Singapore: World Scientific Publishing, 2018.
