# Supplementary material for: Black Rock City versus Manhattan: An economist’s view
Source: PLoS One. 2021 Jan 11;16(1):e0244331. doi: 10.1371/journal.pone.0244331 (PMC7799750; doi:10.1371/journal.pone.0244331)
Supplement: Attachment — Submitted filename: Plos One Response to Reviews.docx [file pone.0244331.s002.docx]

John Yinger

Black Rock City versus Manhattan: An Economist’s View

Resubmission to Plos One

Responses (in Italics) to Reviewers’ Comments

Reviewer #1: The article is well-written. It uses the concrete example of Black Rock City to concretize a new consideration, building upon previous work (e.g., by Anas and Moses). The analysis is sound. Some of it refers to derivations in other works, which is fine. The overall strategy is to demonstrate certain facts using simulation about the difference among aggregates for cities with different street layouts, mainly holding constant either the radius or total population.

Comments:

Section 5 could do with more discussion of the situation in which a planner would be faced with choosing such a design. Black Rock City is a somewhat unusual example in this regard, being rebuilt every year. Mid-century, of course, there were a good number of cities designed and built mostly from scratch, but there are also some in China and India being built today, and the Charter City advocates envision new ones mainly in Latin America and Africa.

It would be illustrative to provide examples of real cities, or parties of real cities, that roughly evoke each class of ideal city.

*These two comments are very helpful. The first version of the paper contained just a few examples of actual cities with the three analyzed street networks. After an extensive internet search, I was able to identify quite a few more examples of all three street network designs. These examples are provided at the beginning of section 5, “Lessons for City Design.”* (See page 17.) *In the previous version, a few examples were mentioned in earlier sections. All these examples (except Black Rock City, Manhattan, and Washington, D.C.) have now been moved to page 17.*

There is a lot of notation. It would be helpful to have a table of notation, for the reader.

*I have added a table of notation to the appendix. This table could be moved to the text if that seems preferable.*

"The objective of this paper is to compare the areas, populations, and average population densities of Circle City, Grid City, and Diag City." I would add something along the lines of "and the maximum distance from the city center to hold a given population." I'm not sure what would be best, but the scope ought to include that the comparisons hold something constant. There are many statements in the paper that could be illuminated by saying what is being held constant as the cities are being compared.

*This is a helpful suggestion. I added two panels to Table 1 indicating the city sizes needed to reach selected city populations. A discussion of the results appears on page 14.*

I think there should be some commentary about the generality of the results. We are proceeding mainly via simulation for a few parameter. This isn't to say it's necessary to prove everything.

*The revised version of the paper adds more emphasis to the point that the simulation results depend on the selected parameters—but the comparisons across cities do not. The two new panels in Table 1 and the revisions to the footnote in Table 1 are directly on this point.*

Reviewer #2: This paper analyzes and compares different city network structures from an economic perspective. While the topic is sound, the paper lacks a clear description of the contributions. The author should better explain the need for each type of the analysis made. In its current form, the paper is difficult to follow. It seems that many parts are build upon the previous studies, thus contributions need to be clearly defined. Since this is an economic analysis, it is useful to incorporate a comprehensive analysis of the transportation cost. There are several studies in the literature comparing different city structures from a public transport perspective that the author might consider incorporating in the paper.

*I agree with the reviewer that the paper should do a better job highlighting its contributions. I have added a comment on Diag City (p. 12) to explain its contribution. I have retained the statements in “lessons” that explain the paper’s other contributions.*

*This paragraph from the reviewer also indicates that the paper does not contain a comprehensive analysis of transportation costs. This is a helpful comment, because it points out a key part of the paper that needs clarification. The street/artery framework is quite general. It includes many types of public transportation systems. Page 5 now has a sentence with examples of possible arteries. More complex transportation networks cannot be analyzed with the type of urban model in this paper. I have added a brief comment on a key example of these issues—commuting time—on page 2. I hope these revisions make the paper clearer.*
